# Dynamic Changes and Regional Differences of Net Carbon Sequestration of Food Crops in the Yangtze River Economic Belt of China

**DOI:** 10.3390/ijerph192013229

**Published:** 2022-10-14

**Authors:** Liping Zhao, Xincheng Li, Xiangmei Li, Chenyang Ai

**Affiliations:** 1 School of Economics, South-Central University for Nationalities, Wuhan 430074, China; 2 Hubei Institute of Building a Well Off Society in an All Round Way, Central South University for Nationalities, Wuhan 430074, China; 3 China Power Engineering Consulting Group, Central Southern Electric Power Design Institute Co., Wuhan 430071, China; 4 Cooperative Innovation Center for Emissions Trading System Co-Constructed by the Province and Ministry, Wuhan 430205, China; 5 School of Low Carbon Economy, Hubei University of Economics, Wuhan 430205, China; 6 College of Business Administration, Zhongnan University of Economics and Law, Wuhan 430073, China

**Keywords:** Yangtze River economic belt (YREB), grain-planting industry, net carbon sequestration, carbon emission, Gini coefficient

## Abstract

The carbon sequestration of food crops is of great significance to slow down agricultural greenhouse gas emissions in agricultural production and management. This paper analyzes the dynamic change and regional differences of net carbon sequestration of food crops from temporal and spatial perspectives for the case study area of the Yangtze River economic belt (YREB) in China. We use the calculation formula of carbon sequestration and carbon emission to calculate the net carbon sequestration in the Yangtze River economic belt. On this basis, we analyze the dynamic trend and regional differences of net carbon sequestration in the Yangtze River economic belt. Furthermore, we use the Gini coefficient to measure the quantitative gap of net carbon sequestration of grain crops in different regions of the Yangtze River economic belt. The results show that: (1) from 2000–2018, the net carbon sequestration of food crops keeps rising within the studied area, while the carbon emission shows a fluctuating downward trend; (2) remarkable regional differences in the net carbon sequestration of food crops have occurred, and most provinces (cities) show an upward trend for the studied area; (3) the unequitable distribution of net carbon sequestration of food crops is clearly displayed in the upper, middle, and lower reaches of the studied area. Moreover, the most uneven place is located on the lower reaches, and the least uneven place is in the upper reaches. These findings are important points of reference for reducing the carbon emissions of the agricultural industry in the Yangtze River economic belt of China and in China more generally.

## 1. Introduction

Nowadays, food security and greenhouse gas emissions are two important issues in agricultural production all over the world [1,2,3]. It is well known that there are two ways to reduce greenhouse gas emissions, i.e., reducing the absolute amount of carbon emissions from the source and increasing carbon sequestration. With regard to food security, scholars pay more attention to the economic value of food crops [4,5] but often ignore the ecological value of food crops [6,7,8]. Food crops can absorb carbon dioxide, regulate the climate, and return farmland soil and straw to the field, which can also fix carbon. Therefore, food crops have an important carbon sequestration function. The carbon sequestration in grain production mainly refers to the carbon absorption in the process of grain growth, which is caused by photosynthesis, while the carbon emission in grain production mainly refers to the emission of greenhouse gases such as CO_2_, CH_4_, and N_2_O on farmlands, which is caused by the input of agricultural materials and the growth of grain crops. The net carbon sequestration in food crops refers to the difference between carbon sequestration and carbon emission. According to relevant scholars [9,10], there are a lot of carbon sequestrations in crop biomass, which can not only increase the organic carbon content of agricultural soil, improve the soil fertility, and increase the grain yield, but they also can reduce the agricultural greenhouse gas emissions and gradually form a valuable ecosystem.

Compared with other sectors, the food production sector is a special sector with dual attributes of carbon source and carbon sequestration. On the one hand, a large number of greenhouse gases are produced in the process of food production, related to the application of petrochemical production such as pesticides and fertilizers. At present, among the carbon dioxide emissions from global human activities, the total carbon dioxide emissions from agricultural production and land-use change account for 24% [11]. In 2017, China accounted for about 29.01% of the total agricultural carbon emissions in Asia and about 12.54% of the total agricultural carbon emissions in the world [12]. On the other hand, the process of crop production can also produce carbon sequestration, offsetting part of the carbon emissions produced from food production itself. That is to say, crops can absorb a large amount of carbon dioxide through photosynthesis during the growth process, which plays a role in purifying the air to a certain extent. As an important agricultural base, China has important contribution to carbon sequestration for food crops [13,14]. Especially, at the general debate of the 75th UN General Assembly, China announced that it would strive to reach the peak of carbon dioxide emissions by 2030 and achieve carbon neutrality by 2060 [15]. Therefore, the study on China’s grain net carbon sequestration is of great significance for achieving the carbon peak and carbon neutrality commitment of the Paris Agreement [16].

Food crops’ carbon sequestration is an important part of agricultural carbon sequestration, and it is very meaningful to study it. At present, the research on food crops’ carbon sequestration is relatively few, and it is mainly focusing on agricultural production. Agricultural carbon sequestration mainly focuses on the following four aspects. Firstly, many scholars have launched the quantitative measurement of agricultural carbon sequestrations. Vleeshouwers et al. [17] constructed a model including climate, agricultural soil, and crops to measure carbon sequestrations in European agricultural soils and found that due to the interaction between crops, soil, and climate, there are great regional differences in the effectiveness of agricultural emission reduction measures and substantial differences in the spatial pattern between carbon sequestrations caused by different measures. Alamdarlo [18] estimated carbon sequestrations in the agricultural sector in the Iranian provinces based on Kuznets and space econometric theory. Dhananjay et al. [19] predicted agricultural carbon emissions in Saskatchewan, Canada, by improving the process-based denitrification decomposition (DNDC) models and concluded that prudent management of agricultural irrigation and fertilizers has a significant impact on enhancing the provincial agricultural carbon sequestration potential. Secondly, the influencing factors of agricultural carbon sequestrations receive much attention, which includes the differences in agricultural systems [20], organic fertilizer inputs and conservation farming [21], land-use change [22], and consumption of agricultural material energy such as straw combustion, feces management, etc. [23,24]. Thirdly, there is research on the agricultural carbon sequestration trading and compensation mechanism. Existing scholars mainly pay attention to ecological compensation issues, compensation principles, compensation methods and standards for agricultural carbon sequestration [25], monitoring, report, and verification (MRV) systems of forest carbon sequestration trading and related systems and policies [26], comparison of similarities and differences in carbon trading across countries [27,28], and so on. Fourthly, it is about the development prospects of agricultural carbon sequestrations. Hoffert et al. [29] consider that changing traditional farming patterns can contribute to reducing agricultural emissions and increasing remittances, especially by implementing conservation farming methods. Ugur et al. [30] used the Granger causal test to explore the relationships between economic growth, agricultural carbon emissions, and energy consumption in Turkey and found that Turkey can promote steady economic growth by effectively reducing agricultural carbon emissions.

A few scholars have studied food crops’ carbon sequestration. She et al. [31] evaluated the carbon inputs and outputs of crop production systems in six typical agricultural regions in China. The results showed that the carbon sequestration of the same crop in different regions was significantly different, as well as different crops in the same region. Among the three major crops in China, the total annual net carbon sink of rice was the highest. Kang et al. [32] analyzed the impact of grain production on ecological carbon sink in China. Research shows that grain production helps to increase ecological carbon sink. Compared with northeast and western regions, the carbon sink effect of grain production in eastern and central regions is greater.

In summary, scholars have carried out a lot of research on measuring agricultural carbon sequestration, influencing factors of carbon sequestration, the carbon sequestration trading and compensation mechanism, carbon sequestration development prospects, etc.

However, from the perspective of research object, the existing research mainly takes forest carbon sequestration as the research object, and there is less research taking food crops as the research object; from the perspective of research content, the research about carbon sequestration rarely considers the impact of carbon emission. Therefore, based on the calculation of the carbon sequestration and carbon emission of grain crops, taking grain crops as the research object, the net carbon sink as the research content, and the Yangtze River economic belt (YREB) as a case study area, this paper analyzes the dynamic changes and regional differences of net carbon sequestration of grain crops in different regions of China’s Yangtze River economic belt.

This paper has three specific objectives to obtain: (1) to estimate the net carbon sequestration of grain crops from two aspects, i.e., carbon sources and carbon sequestrations; (2) to analyze the dynamic trend and regional differences of net carbon sequestration; (3) to measure the quantitative gap of net carbon sequestration of grain crops in different regions by using the Gini coefficient.

## 2. Materials and Methods

### 2.1. Site Description

**Figure 1:**
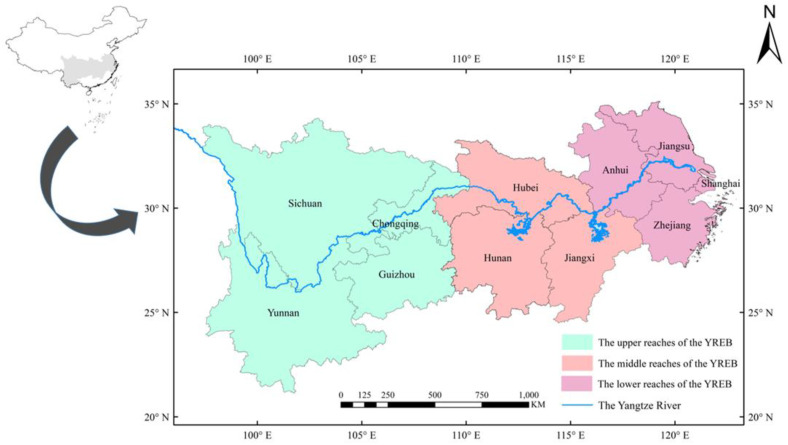
Location map of Yangtze River economic belt.

The Yangtze River economic belt (YREB) lies between 20°–35° N and 90°–122° E, covering the three major regions of east, middle, and west in China, which consist of eleven provinces (cities), including Shanghai, Jiangsu, Zhejiang, Anhui, Jiangxi, Hubei, Hunan, Chongqing, Sichuan, Yunnan, Guizhou, etc. (Figure 1). Its area is about 2,052,300 km^2^, accounting for 21.4% of total area of China. Moreover, its population and gross production value all surpass 40% of that of China. The YREB can be divided into upper reaches, middle reaches, and lower reaches. The upper reaches include Chongqing, Sichuan, Guizhou, and Yunnan, the middle reaches include Jiangxi, Hubei, and Hunan, and the lower reaches include Shanghai, Jiangsu, Zhejiang, and Anhui. In November 2018, Chinese government fully exerted the location advantage of the YREB, guided by ecological priority and green development, to promote the coordinated development of the upper, middle, and lower reaches of the Yangtze River and the high-quality development of the regions along the Yangtze River.

The YREB is an important food production base in China. It is abundant in natural resources, especially arable land, which together cover one third of the total area of China. In addition, the output value of agriculture, forestry, animal husbandry, and fishery accounts for about 40% of that of China. The YREB consists of several plains, such as Jianghan Plain in Hubei province, Dongting Lake Plain in Hunan province, Chengdu Plain in Sichuan province, Poyang Lake Plain in Jiangxi province, and Taihu Plain in Jiangsu and Zhejiang province and other country-level commodity grain bases. Its grain production accounts for about 40% of the country.

Based on the differences in conditions of geography, soil, climate, technology, etc., as well as the total amount of food production and consumption in different regions of the studied area, China is divided into three regions, i.e., the main grain-producing area, the main grain marketing area, and the grain balance area. Main grain-producing area refers to key grain production areas that have suitable natural resource conditions for cultivating food crops and have certain technical advantages and economic effects. The main grain marketing area refers to the grain consumption area with more people and less land, low food self-sufficiency with rate of grain, and large gap in grain production and demand, which is mainly distributed in economically developed areas, such as the southeast coast of China and large cities. The grain balance area refers to the western region of China which is mainly located in remote areas with relatively backward economy, self-sufficiency in food production, and basic balance between production and demand in food.

The YREB has six main grain-producing areas, two main grain marketing areas, and three grain balance areas. The six main grain-producing areas are distributed in Sichuan, Hubei, Hunan, Jiangsu, Jiangxi, and Anhui provinces. The two main grain marketing areas are located on Zhejiang province and Shanghai city. The three grain balance areas are situated in Yunnan, Guizhou provinces, and Chongqing city. The Chinese government has committed to building the YREB as a good ecological environment and high-quality development economic belt.

As the planting industry is the main agricultural area type in the studied area, exploring the net carbon sequestration of grain-planting industry is of great significance to promote the green transformation of grain production and protect the agricultural ecological environment in the studied area.

### 2.2. Research Methods

#### 2.2.1. Research Framework

**Figure 2:**
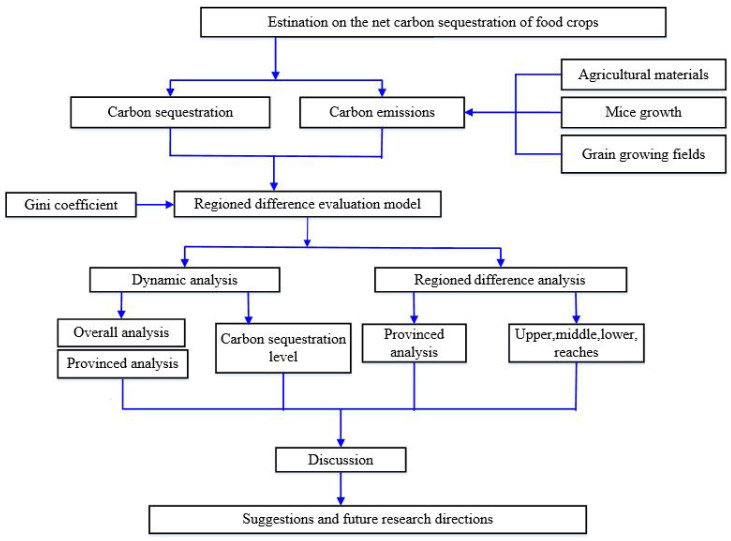
Research framework.

In this paper, the net carbon sequestration of food crops is estimated in the following steps (as shown in Figure 2). First, it is clarified that the net carbon sequestration is influenced by both carbon sequestration and carbon emission (consisting of three components: agricultural materials, rice growth, and grain growing fields), and on this basis, a regional difference evaluation model is constructed using the Gini coefficient. Then, this paper analyzes the dynamic change and regional differences of net carbon sequestration of food crops for the case study area of the YREB in China. In terms of time, this paper separately analyzes the dynamic change trend of net carbon sequestration in the whole study area, and based on this, the net carbon sequestration and the level of carbon sequestration of food crops in 11 provinces (cities) were analyzed dynamically. In terms of space, through the Gini coefficient, this paper analyzes provincial differences of the net carbon sequestration level in the whole study area and the regional differences of the net carbon sequestration level in in the upper, middle, and lower reaches of the YREB. Finally, the results of this paper are discussed, and feasible suggestions and future research directions are given.

#### 2.2.2. Estimation on the Carbon Sequestration of Food Crops

The net carbon sequestration of food crops is the difference between carbon sequestration and carbon emission, and the carbon sequestration level of food crops is the ratio of carbon sequestration to carbon emission. If the value is greater, the capacity of the net carbon sequestration is stronger, which can better reflect the effect of the net carbon sequestration in a region. The carbon sequestration of food crops was calculated as follows:

(1) $$C_{t}=\overset{k}{\underset{i=1}{\sum }}C_{i}=\overset{k}{\underset{i=1}{\sum }}r_{i}\cdot y_{i}\cdot \left(1-w_{i}\right)/EC_{i}$$

where $\;C_{t}\;$ represents the total carbon absorption of food crops; *i* is the type of food crops; $C_{i}$,$\;r_{i}$, $y_{i\;}$,$\;w_{i}$, and $EC_{i\;}$ are the carbon absorption, the carbon absorption rate, the economic yield, water content, and economic coefficient of certain food crops, respectively. The carbon absorption rate, water content, and economic coefficient of various food crops are from the Intergovernmental Panel on Climate Change (IPCC) report [33] (Table 1).

#### 2.2.3. Estimation on the Carbon Emission of Food Crops

The carbon emission of food crops consists of three parts. The first part comes from the input of agricultural materials, which mainly include chemical fertilizer, pesticide, agricultural film, plastic film, agricultural diesel, and agricultural land irrigation, etc. Here, we apply the weight coefficient A to separate the input of the food production from the generalized input of agricultural production. Especially, the weight coefficient A = the area of food crops sowing/the area of crops sowing. The second part originates from CH_4_ emissions caused by the rice growth. The emission factor is taken as a comprehensive emission factor that takes into account regional differences, climate change, etc., which is more convincing and more realistic than a single emission coefficient, as shown in Table 2. The relevant coefficients are recommended by the report of IPCC [33]. The third part comes from N_2_O emissions caused by grain growing fields [33]. As such, the carbon emission of food crops can be calculated with following equation:

(2) $$E_{t}=\sum_{i=1}^{k}E_{i}=\sum_{i=1}^{k}T_{i}\cdot \delta_{i}$$

where i is the type of carbon sources; k is the number of carbon sources; $E_{t}$ is the total carbon emission from the food crops; $E_{i}$ is the amount of carbon emission from each carbon source; $T_{i}$ is the amount of each carbon source; $\delta_{i}$ is the carbon emission coefficient of each carbon source.

#### 2.2.4. Construction of Regional Difference Evaluation Model of Net Carbon Sequestration

Gini coefficient is an important index that can measure the inequality of income and wealth distribution, which can comprehensively investigate the difference of income distribution among residents. As such, this paper uses Gini coefficient to measure the regional distribution fairness of carbon sequestration from food crops in the YREB and further to investigate the regional differences of net carbon sequestration of food production. Here, we propose a hypothesis: if the proportion of carbon sequestration from food crops of each province (city) in the overall region is completely consistent with the proportion of carbon emission from food crops of one, there is absolute fairness among the regional distributions of net carbon sequestration in the YREB.

**Figure 3:**
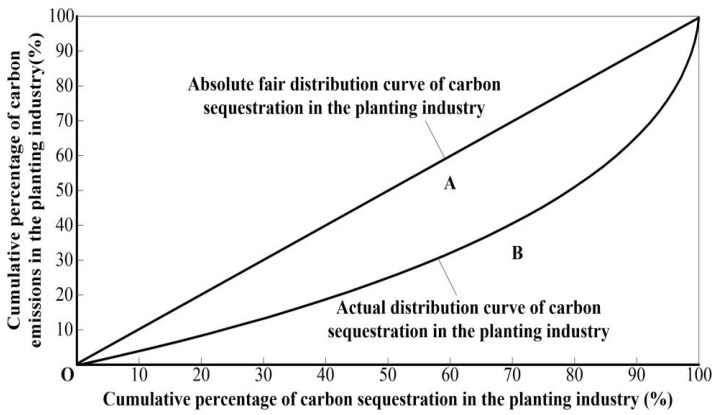
Lorentz curve diagram of net carbon sequestration.

Otherwise, we consider there are regional differences. From Figure 3, A represents the area between the absolute average distribution curve of carbon sequestration and the actual distribution curve of carbon sequestration. B is the area between the actual distribution curve of carbon sequestration and horizontal axis. The Gini coefficient of net carbon sequestration from food crops is A/(A + B).

Generally, the Gini coefficient is between 0 and 1. The smaller the Gini coefficient is, the more even the distribution is. On the contrary, the larger the Gini coefficient is, the more uneven the distribution is. Based on the value of Gini coefficient, we can obtain five types, i.e., absolute average, relatively average, relatively reasonable, large gap, and wide gap, which are represented by below 0.2, between 0.2 and 0.3, between 0.3 and 0.4, between 0.4 and 0.5, and more than 0.5, respectively [34]. Moreover, 0.4 is usually regarded as the “warning line” of income gap. According to this international standard, the distribution equity of net carbon sequestration from food crops is calculated with following equation:

(3) $$Ginicoefficient=1-\sum_{j=1}^{n}\left(X_{j}-X_{j-1}\right)\left(Y_{j}+Y_{j-1}\right)$$

where j is the province; $\;X_{j}$ is the cumulative percentage of carbon sequestration from food crops; $Y_{j}$ is the cumulative percentage of carbon emissions from food crops. When j = 1, $X_{j-1}$, and $Y_{j-1}$ are regarded as 0, the horizontal axis represents the cumulative percentage of carbon sequestration from food crops of each province (city) in the overall region. When the proportion of carbon sequestration from food crops in certain province (city) is greater than that of carbon emission, it means that the ecological environment of this province (city) is good for food production. Meanwhile, this indicates the region has higher ecological capacity and can share part of the carbon emission from food crops for other provinces (cities).

### 2.3. Data Sources

This paper takes the five kinds of main food crops as the research objects, i.e., rice, wheat, corn, beans, and tubers, and the time range is 2000–2018.

For the indicator of the carbon sequestration of food crops, the data on the carbon absorption, the carbon absorption rate, the economic yield, water content, and economic coefficient of certain food crop are from the IPCC report, and the data on the economic yield are from the *China Rural Statistical Yearbook*; for the indicator of the carbon emission of food crops, the data on the output of various food crops, the sowing area of the rice, the amount of chemical fertilizer, pesticide, agricultural film, plastic film, agricultural diesel, and the agricultural land irrigation invested in the food production are from the *China Rural Statistical Yearbook,* and the data on the CH_4_ emissions caused by the rice growth and the N_2_O emissions caused by grain growing fields are from the IPCC report.

## 3. Results and Analysis

### 3.1. Dynamic Analysis on the Net Carbon Sequestration of Food Crops

#### 3.1.1. Overall Dynamic Analysis on the Net Carbon Sequestration of Food Crops

**Figure 4:**
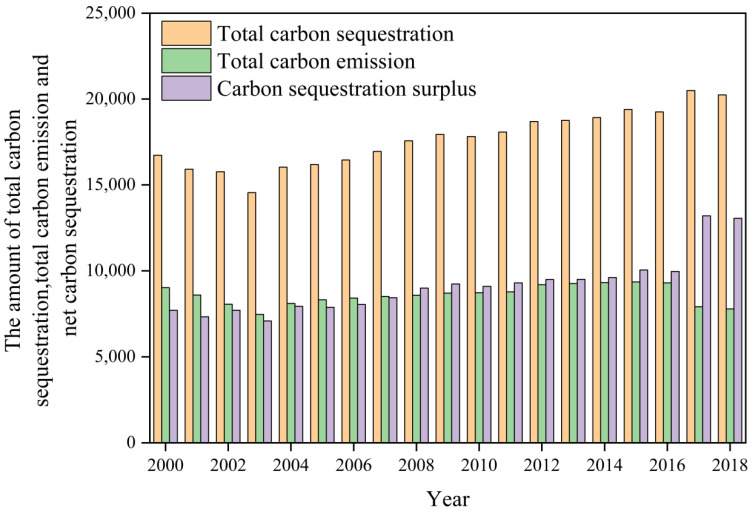
The total carbon sequestration, total carbon emission, and net carbon sequestration from food crops in the YREB (10^4^ t C).

The total amount of the carbon sequestration, carbon emission, and net carbon sequestration from food crops in the YREB from 2000–2018 is shown in Figure 4. The net carbon sequestration of food crops maintains an upward trend, while the carbon emission shows a fluctuating downward trend. From 2000–2018, the net carbon sequestration of the region increases from 7.70669 × 10^7^ t to 1.306066 × 10^8^ t, which increases by 69.47%. The carbon emission decreases from 9.02022 × 10^7^ t to 7.78215 × 10^7^ t, which decreases by 15.09%. The total carbon sequestration increases from 1.672690 × 10^8^ t to 2.024332 × 10^8^ t, which increases by 21.02%. On the whole, the carbon sequestration from food crops in the YREB is gradually increasing. The carbon emission shows a “decline-rise-decline” trend, which indicates that the ecological environment of the food production in the YREB is gradually improving.

#### 3.1.2. Dynamic Analysis on the Net Carbon Sequestration of Food Crops in Various Provinces (Cities)

**Figure 5:**
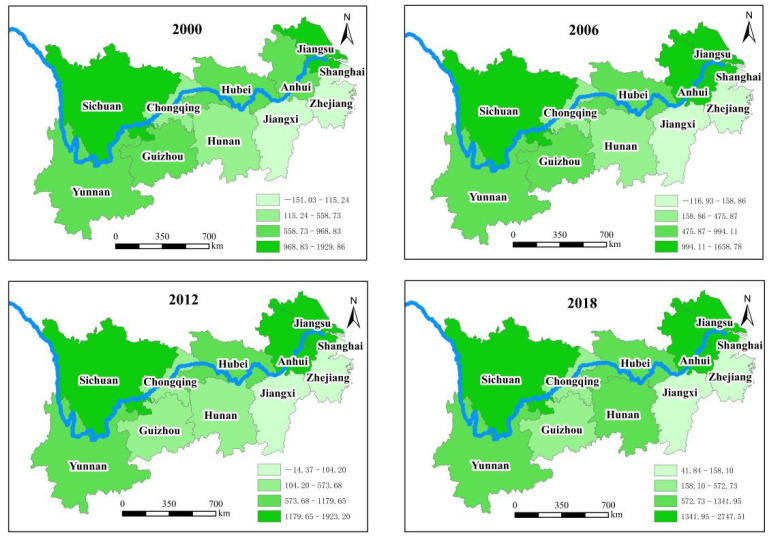
Changes of total net carbon sequestration of food crops in 11 provinces (cities) of YREB.

The levels of net carbon sequestration of food crops in various provinces (cities) in the YREB are shown in Figure 5. Here, we only show the results of 2000, 2006, 2012, and 2018 in Figure 5. The detailed results are presented in the Supplementary File (Table S1). Compared with 2000, the net carbon sequestration of food crops in various provinces (cities) in 2018 shows obvious regional differences. Except for Shanghai and Guizhou, the other nine provinces (cities) show an upward trend. Especially, Guizhou as a grain balance area shows the largest decline rate of 18.88%. In addition, Shanghai as a main grain sales area has a decline rate of 12.19%. The possible reason is that the grain sown area has been greatly reduced with the transformation of industrial structure in Guizhou and Shanghai, which leads to a decrease of carbon sequestration accordingly. According to the *China Statistical Yearbook*, the grain-sowing area of Guizhou falls from 3153.3 thousand hm^2^ to 2740.2 thousand hm^2^ from 2000 to 2018, and the grain-sowing area of Shanghai falls from 258.8 thousand hm^2^ to 133.1 thousand hm^2^. In addition, the rising range of the net carbon sequestration in Anhui is the largest, which increases by 183.59%, which is followed by Hunan and Jiangxi, i.e., 171.67% and 137.87%, respectively. Although the ranking of net carbon sequestration in Hubei and Jiangsu province in 2018 is not at the top, with the gradual increase of grain-sowing area, the net carbon sequestration is also increasing gradually.

Taking 2018 as a horizontal comparison year, the net carbon sequestration of food crops in 11 provinces (cities) of the YREB is ranked as follows: Anhui > Jiangsu > Sichuan > Yunnan > Hubei > Hunan > Guizhou > Chongqing > Zhejiang > Jiangxi > Shanghai. Five provinces in the major grain-producing area are at the top of the ranking, i.e., Anhui, Jiangsu, Sichuan, Hubei, and Hunan. Two provinces in the main grain sales area list in the lower ranking, i.e., Shanghai and Zhejiang, and two provinces in the grain balance area are in the middle of the ranking, i.e., Yunnan and Guizhou. We demonstrate that the main grain-producing area receives more attention to the protection of grain ecological environment, compared with the main grain sales area and the grain balance area. The gap of net carbon sequestration of food crops is very large in the major grain-producing area, i.e., 2.74751 × 10^7^ t for Anhui, 2.24578 × 10^7^ t for Jiangsu, only 1.33197 × 10^7^ t for Hubei, and 1.15972 × 10^7^ t for Hunan, respectively. The possible reason is that Anhui and Jiangsu have relatively advanced agricultural production technology and production concept, which results in a relatively good agricultural ecological environment.

### 3.2. Dynamic Analysis on the Carbon Sequestration Level of Food Crops in Various Provinces (Cities)

**Figure 6:**
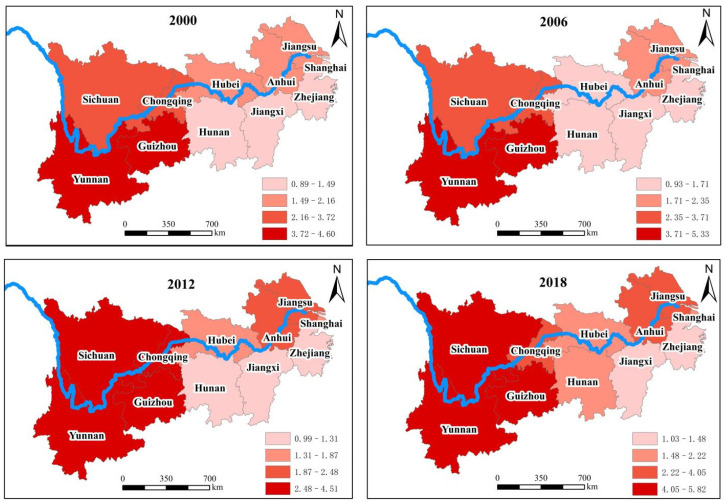
Changes of carbon sequestration level of food crops in 11 provinces (cities) of YREB. (Note: The level of carbon sequestrations is the ratio between carbon sequestrations and carbon emissions).

The carbon sequestration levels of food crops in various provinces (cities) are shown in Figure 6. The carbon sequestration level of food crops in 2018 is ranked as follows: Yunnan > Sichuan > Guizhou > Chongqing > Anhui > Jiangsu > Hubei > Shanghai > Hunan > Zhejiang > Jiangxi. Compared with the ranking of net carbon sequestration, the ranking of Yunnan, Sichuan, and Guizhou is relatively at the top, while that of Jiangsu, Hubei, and Hunan lags behind. In addition, the rising range of Anhui is the largest, attaining 90.59%. Correspondingly, the rising range of Guizhou is the smallest, attaining only 90.59%.

According to the time series changing characteristics of carbon sequestration level in each province (city), we can divide 11 provinces (cities) into three types of areas, i.e., the continuous growth area, the fluctuating growth area, and the fluctuating descent area. For the continuous growth area, the level of carbon sequestration shows an increasing trend at each time point compared with the previous time point. As such, six provinces (cities) such as Anhui, Jiangsu, Chongqing, Jiangxi, Hubei, and Hunan meet this characteristic. For the fluctuating growth area, the level of carbon sequestration shows an upward trend on the whole and a downward trend at some time points. As such, three provinces such as Zhejiang, Sichuan, and Yunnan meet this characteristic. For the fluctuating descent area, the level of carbon sequestration shows a downward trend on the whole and an upward trend at some time points. As such, two provinces (cities) such as Shanghai and Guizhou meet this characteristic.

### 3.3. Regional Difference Analysis on the Net Carbon Sequestration of Food Crops

In this paper, the regional differences of the net carbon sequestration level of food crops are analyzed in the upper, middle, and lower reaches of the YREB.

#### 3.3.1. Regional Differences of Net Carbon Sequestration Level of Food Crops in 11 Provinces (Cities) of the YREB

**Figure 7:**
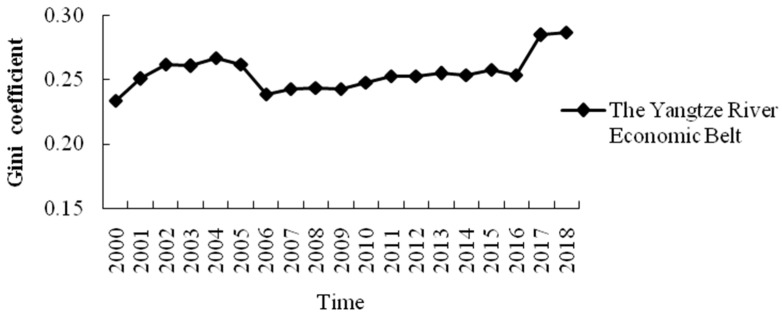
The Gini coefficient of the net carbon sequestration level of food crops in the 11 provinces (cities) of the YREB.

From Figure 7, the Gini coefficient of the net carbon sequestration level of food crops in 11 provinces (cities) shows an upward trend overall from 0.234 in 2000 to 0.287 in 2018, which increases by 22.65% during the studied period. It shows that the regional difference of the net carbon sequestration level of food crops in various provinces (cities) is gradually expanding. The Gini coefficient value indicates that the distribution characteristics of the net carbon sequestration level in various provinces (cities) are in the state of “relatively average” from 2000–2018. Especially, the regional difference of net carbon sequestration level in various provinces (cities) became smaller from 2006–2016, while becoming wider after 2016.

#### 3.3.2. Regional Differences of Net Carbon Sequestration Level of Food Crops in the Upper, Middle, and Lower Reaches of Yangtze River Economic Belt

**Figure 8:**
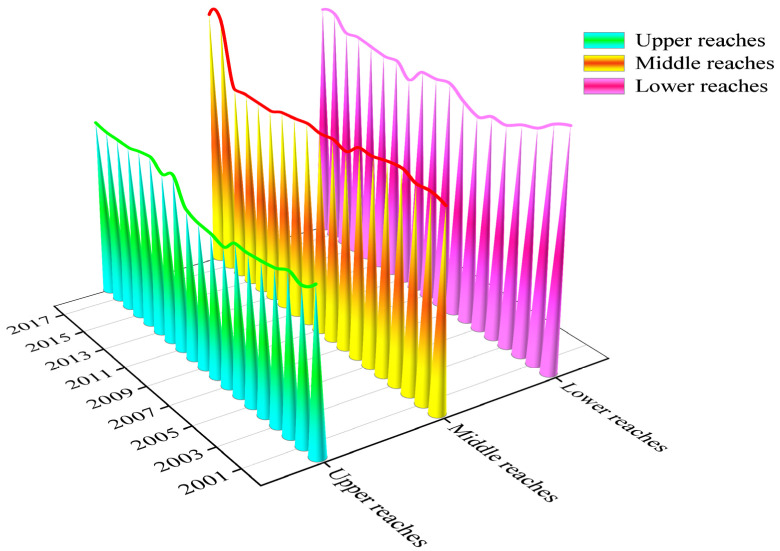
Evolution of intra-regional gap of net carbon sequestration of grain food crops in the YREB.

As is shown in Figure 8, the values of the Gini coefficient in the upper, middle, and lower reaches of the YREB are ranked as follows: lower reaches > middle reaches > upper reaches. This indicates that the regional difference in the lower reaches is the largest, followed by the middle reaches and the upper reaches, which may be caused by the differences of food production in various provinces (cities). Taking the lower-reaches region as an example, the food-sowing area in Zhejiang and Shanghai is relatively small and declining year by year. The food-sowing area in Jiangsu and Anhui is gradually increasing, from 5304.3 thousand hm^2^ to 5475.9 thousand hm^2^ in Jiangsu and from 6183.8 thousand hm^2^ to 7316.3 thousand hm^2^ in Anhui during the studied period. The regional difference of the net carbon sequestration level in the middle reaches is relatively small, which is attributed to the small difference of the food-sowing area.

From Figure 8, the Gini coefficient value in the upper-reaches region is between 0.171 and 0.218, which indicates the distribution characteristic of the net carbon sequestration level changes from the “absolute average” state to the “comparative average” state, showing a fluctuating upward trend. Moreover, the value of the Gini coefficient in 2007 is the smallest, which indicates that the regional difference of the distribution of net carbon sequestration was the smallest. In 2013, the value of Gini coefficient reached the maximum value, which signifies that the regional difference of the distribution of net carbon sequestration was the largest.

The values of the Gini coefficient for food crops in the lower reaches are between 0.252 and 0.293, which indicates the distribution characteristics are in a “comparative average” state. The change trend is similar with that in the middle reaches. However, the regional difference of the net carbon sequestration is larger than that in the middle-reaches region, which generally presents a “decline-rise-decline-rise” state. In 2006, the Gini coefficient reached the minimum value, which indicates that the regional difference of net carbon sequestration in 2006 was the smallest. In 2000, the Gini coefficient reached the maximum value, so the regional difference of the distribution of net carbon sequestration in 2000 was the largest.

The values of Gini coefficient in the middle reaches are between 0.235 and 0.309, which indicates the distribution of net carbon sequestration changed from the “comparative average” state to the “relatively reasonable” state. The regional distribution difference of the middle reaches is between the upper reaches and lower reaches, and the change trend shows a “decline-up” state. In 2005, the Gini coefficient reached the minimum, so the regional difference of the distribution of net carbon sequestration in 2005 was the smallest. In addition, the Gini coefficient reached the maximum value in 2017, which indicates the regional difference of the distribution of net carbon sequestration in 2017 was the largest.

## 4. Discussion

### 4.1. The Spatio-Temporal Patterns of Net Carbon Sequestration of Food Crops in the YREB

The net carbon sequestration of food crops in the YREB experiences two different stages from declining to increasing over the study period. In the first stage (2000–2003), the net carbon sequestration continued to decline; this is probably because farmers’ enthusiasm for planting the food crops is reduced by the heavy tax burden, which directly affected the production of rice, wheat, and maize, resulting in a decline in the net carbon sequestration of food crops during this period. In the second stage (2004–2018), the net carbon sequestration was on a rising trend. Since 2004, the agricultural tax has been abolished in China, which promoted the increase of grain-sowing areas, and the total output of grain in the Yangtze River economic belt continued to grow, resulting in the continuous increase of net carbon sequestration of food crops.

Understanding and analyzing the heterogeneous heterogeneity characteristics of the net carbon sequestration level in different areas’ provinces and cities contributes to establishing the corresponding emission reduction concept. The net carbon sequestration of food crops in various provinces (cities) shows obvious regional difference from 2000 to 2018; except for Shanghai and Guizhou, the other nine provinces (cities) show an upward trend. The carbon sequestration of food crops in the main grain-producing areas is higher than that in other areas, from which can be inferred that the provinces in the main grain-producing areas pay more attention to the protection of environment.

The petrochemical agriculture that relies on the input of chemical fertilizers and pesticides contributes to the increase of grain production in a certain period time, but it also brings great damage to the environment such as water resources, arable land, and the atmosphere, as well as food quality. As such, the green transformation needs to be implemented to increase the organic matter content of soil, improve the quality of water resources and air, and finally ensure food safety. At the same time, technicians should perform demonstrations and training of green conservation farming technology, such as straw returning, green control of diseases and insect pests, soil testing formulas, scientific fertilization, and medicine, so as to reduce carbon emissions from food production at the source.

### 4.2. The Regional Differences of Net Carbon Sequestration of Food Crops in the YREB

The values of the Gini coefficient indicate that the regional difference in the lower reaches is the largest, followed by the middle reaches and the upper reaches. The main reason for the above differences may lie in the differences of grain-sowing areas in the upper, middle, and lower reaches of the Yangtze River economic belt. Among all regions, the level of economic development in the lower reaches is the highest, and with the increase of the urbanization rate, a large amount of agricultural land has been occupied by industrial land; thus, the area of cultivated land has gradually shrunk in some provinces, such as in Zhejiang and Shanghai. As a result, the difference of grain-sown areas in this region is growing. However, there are many mountains in the downstream area; thus, there is less arable land in some provinces, such as Yunnan and Guizhou, so there is also a certain difference in this region. The provinces in the middle reaches are mainly the main grain-producing provinces, so the difference of grain-sown areas in this region is relatively small.

To avoid the above problems, the government should carry out the following tactics, including the expansion of the sown area, curbing the non-agricultural conversion of arable land, improving the multiple cropping index of the arable land, increasing the investment in grain breeding technology, speeding up the modernization of the entire grain industry chain, etc., so as to expand the sown area of food, increase the carbon sequestration of food crops, and finally reduce the regional differences of net carbon sequestration of food crops in the YREB. In addition, downstream provinces should also give some ecological compensation to midstream provinces to reward their contributions to food carbon sequestration.

### 4.3. Advantages and Limitations of This Study and Future Research Directions

Compared with previous studies, the research vision of this study is further expanded. This study develops an objective framework for evaluating the environmental benefits of grain planting from the perspective of net carbon sequestration and analyzes the spatial and temporal characteristics of the net carbon sequestration in different areas. It is no longer limited to the single-perspective research of agricultural carbon emission, and it conducts research from the two perspectives of agricultural carbon emission and agricultural carbon sequestration, which can better analyze the environmental problems of agricultural production. In this study, the concept of net carbon sequestration is used to analyze carbon sequestration and carbon emission in the same framework.

However, there are also some limitations in this study. Firstly, there are some limitations in the definition and calculation system of carbon emission, carbon sequestration, and net carbon sequestration, which need to be further refined and improved. Secondly, due to the limitations of the existing data, we apply the same indicators in different areas of the YREB, where they may have different features such as economic coefficient, water content, and carbon absorption in the upper, middle, and lower reaches. Thirdly, factors such as population growth, the industrialization rate, and other influencing factors also have an impact on the carbon sequestration of food production. However, the existing methods for measuring carbon emissions and carbon sequestration of food crops mainly consider the internal factors related to the growth of food crops and do not consider the external factors such as population and industrialization rate. In future research, we will use econometric research methods to analyze the impact of population, industrialization rate, and other influencing factors on carbon emissions and carbon sequestration of food crops. In addition, this study does not analyze the economic benefits of net carbon sequestration of food crops, which will be taken into consideration in future studies.

## 5. Conclusions

This study analyzes the dynamic change and regional differences of net carbon sequestration of food crops from temporal and spatial perspectives for the case study area of 11 provinces (cities) of the YREB from 2000–2018 in China. The net carbon sequestration of food crops keeps continuously increasing, while carbon emissions show a fluctuating downward trend over the study period. Remarkable regional differences in the net carbon sequestration of food crops exist, and most provinces (cities) show an upward trend for the studied area. Except for Shanghai and Guizhou, the remaining nine provinces (cities) show an upward trend, and the decline range of Guizhou is the largest. The spatial distributions of the net carbon sequestration of food crop show obvious heterogeneity in the upper, middle, and lower reaches of studied area. Specifically, the Gini coefficient value in the YREB is ranked as: lower reaches > middle reaches > upper reaches. That is to say that the most uneven place is located on the lower reaches, followed by the middle reaches, and the least uneven place is in the upper reaches. To further facilitate the activity related to reducing the carbon emissions of the agricultural production sector, we will explore the economic benefits and the influencing factors of net carbon sequestration from food production in the future.

## Figures and Tables

**Table 1:** Economic coefficients, water content, and carbon absorption rates of major food crops in China.

Category	Economic Coefficient	Water Content/%	Carbon Absorption Rate
Rice	0.45	12	0.414
Wheat	0.40	12	0.485
Corn	0.40	13	0.471
Beans	0.34	13	0.450
Tubers	0.70	70	0.423

**Table 2:** The CH_4_ emission coefficient of rice during its growth cycle in different regions of Yangtze River economic belt (g/m^2^).

Region	Early Rice	Late Rice	Mid-Season Rice	Region	Early Rice	Late Rice	Mid-Season Rice
Shanghai	12.41	27.5	53.87	Hunan	14.71	34.1	56.28
Jiangsu	16.07	27.6	53.55	Chongqing	6.55	18.5	25.75
Zhejiang	14.37	34.3	57.96	Sichuan	6.55	18.15	25.73
Anhui	16.75	27.6	51.24	Guizhou	5.1	21	22.05
Jiangxi	15.47	45.8	65.42	Yunnan	2.38	7.6	7.25
Hubei	17.51	39	58.17				

## Data Availability

Not applicable.

## Supplementary Materials

The following are available online at https://www.mdpi.com/article/10.3390/ijerph192013229/s1, Table S1: Changes of total net carbon sequestration and carbon sequestration level of food crops in 11 provinces (cities) of Yangtze River economic belt (2000–2018).
