# Supplementary material for: Dynamic Changes and Regional Differences of Net Carbon Sequestration of Food Crops in the Yangtze River Economic Belt of China
Source: Int J Environ Res Public Health. 2022 Oct 14;19(20):13229. doi: 10.3390/ijerph192013229 (PMC9602910; doi:10.3390/ijerph192013229)
Supplement: Supplementary file 1 [file ijerph-19-13229-s001.zip › ijerph-1894332-supplementary.pdf]

Supplementary Saterials: Supplementary data

Table S1. Changes of totalnet carbon sequestration and carbon sequestration level of food crops in 11 provinces (cities) of Yangtze River Economic Belt (2000-2018).

Region	2000		2001		2002		2003		2004		2005		2006		2007		2008		2009	
	Carbon sequestration level	Carbon sequestration surplus	Carbon sequestration level	Carbon sequestration surplus	Carbon sequestration level	Carbon sequestration surplus	Carbon sequestration level	Carbon sequestration surplus	Carbon sequestration level	Carbon sequestration surplus	Carbon sequestration level	Carbon sequestration surplus	Carbon sequestration level	Carbon sequestration surplus	Carbon sequestration level	Carbon sequestration surplus	Carbon sequestration level	Carbon sequestration surplus	Carbon sequestration level	Carbon sequestration surplus
Shanghai	1.49	47.65	1.46	39.17	1.46	33.80	1.49	26.28	1.53	30.16	1.48	27.83	1.57	33.18	1.55	32.15	1.62	36.75	1.71	42.05
Jiangsu	2.16	1445.16	2.20	1378.93	2.26	1382.15	2.11	1111.82	2.18	1318.11	2.10	1280.07	2.22	1448.90	2.27	1549.08	2.31	1596.58	2.34	1636.64
Zhejiang	1.14	115.24	1.16	114.63	1.21	128.52	1.24	119.29	1.23	121.58	1.19	101.13	1.30	158.86	1.15	73.70	1.22	111.09	1.24	118.67
Anhui	1.84	968.83	2.05	1116.17	2.28	1333.07	1.93	913.97	2.26	1328.77	2.13	1208.06	2.35	1477.78	2.36	1551.25	2.46	1674.46	2.47	1700.46
Jiangxi	0.89	-151.03	0.90	-148.37	0.88	-169.00	0.87	-167.98	0.88	-172.28	0.90	-147.88	0.93	-116.93	0.95	-75.56	0.96	-60.28	0.98	-38.46
Hubei	1.69	735.97	1.64	671.47	1.67	661.25	1.70	636.54	1.71	700.08	1.70	727.32	1.71	748.39	1.77	809.89	1.80	841.29	1.81	876.70
Hunan	1.24	426.89	1.27	452.66	1.24	373.87	1.28	424.65	1.28	452.78	1.26	438.68	1.29	475.87	1.24	420.78	1.29	499.71	1.30	534.29
Chongqing	3.20	558.73	2.90	477.53	3.54	558.72	3.78	578.98	3.93	614.23	4.04	634.86	3.31	473.19	4.16	600.74	4.19	622.96	4.14	619.54
Sichuan	3.72	1929.86	3.28	1591.29	3.88	1821.39	4.01	1792.94	4.08	1864.15	4.10	1899.01	3.71	1658.78	3.90	1816.25	4.05	1903.91	4.06	1915.12
Guizhou	4.20	706.00	3.97	650.46	4.16	615.16	4.61	674.17	4.83	709.98	4.79	708.80	4.70	686.13	4.78	678.81	4.81	712.98	4.79	719.77
Yunnan	4.60	923.38	4.67	982.09	5.29	966.16	5.53	972.39	5.36	966.75	5.47	996.06	5.33	994.11	5.26	977.44	5.45	1056.61	5.59	1109.31

Region	2010		2011		2012		2013		2014		2015		2016		2017		2018	
	Carbon sequestration level	Carbon sequestration surplus	Carbon sequestration level	Carbon sequestration surplus	Carbon sequestration level	Carbon sequestration surplus	Carbon sequestration level	Carbon sequestration surplus	Carbon sequestration level	Carbon sequestration surplus	Carbon sequestration level	Carbon sequestration surplus	Carbon sequestration level	Carbon sequestration surplus	Carbon sequestration level	Carbon sequestration surplus	Carbon sequestration level	Carbon sequestration surplus
Shanghai	1.70	40.52	1.79	45.25	1.77	43.86	1.72	39.00	1.59	33.81	1.75	39.39	1.61	30.87	1.83	37.50	1.93	41.84
Jiangsu	2.34	1643.19	2.38	1703.63	2.37	1728.87	2.40	1776.54	2.39	1765.77	2.47	1875.21	2.42	1809.87	3.04	2222.14	3.04	2245.78
Zhejiang	1.22	109.31	1.26	128.06	1.21	104.20	1.15	75.91	1.16	80.35	1.17	83.45	1.19	94.47	1.47	149.14	1.48	158.10
Anhui	2.46	1706.66	2.49	1753.79	2.48	1845.01	2.44	1828.97	2.46	1853.93	2.57	2012.58	2.53	1963.76	3.61	2769.14	3.59	2747.51
Jiangxi	0.94	-95.06	0.98	-27.30	0.99	-14.37	1.00	2.60	1.02	26.84	1.01	21.81	1.02	27.57	1.58	652.56	1.03	57.20
Hubei	1.83	888.39	1.87	943.53	1.87	967.24	1.86	986.95	1.85	999.72	1.91	1081.91	1.87	1013.24	2.23	1349.16	2.22	1331.97
Hunan	1.28	497.68	1.31	558.70	1.31	573.62	1.27	503.11	1.30	552.81	1.31	563.52	1.30	539.87	1.89	1171.49	1.91	1159.72
Chongqing	4.20	630.55	4.07	610.76	3.40	573.42	3.41	575.96	3.38	574.24	3.42	573.11	3.45	580.49	4.01	571.17	4.05	571.88
Sichuan	4.11	1940.81	4.17	1984.22	3.75	1923.20	3.85	1981.56	3.83	1966.74	3.84	1985.06	3.92	2035.69	4.89	2219.68	4.99	2232.49
Guizhou	4.61	685.28	3.23	426.85	3.51	573.68	3.27	506.73	3.40	542.77	3.63	592.20	3.73	614.64	5.36	762.10	4.69	572.73
Yunnan	5.14	1046.52	5.45	1173.07	4.51	1179.65	4.53	1221.62	4.45	1212.42	4.41	1216.32	4.47	1249.41	5.12	1292.96	5.82	1341.95
